# A consensus-based treatment algorithm for alopecia areata in adolescents and adults: integrating severity, quality of life, and disease dynamics

**DOI:** 10.3389/fmed.2026.1846243

**Published:** 2026-07-15

**Authors:** Charles W. Lynde, Lyn Guenther, Anneke Andriessen, Sameh Hanna, Elena Netchiporouk, Vimal Prajapati, Marni Wiseman, Julien Ringuet

**Affiliations:** 1 Lynde Institute for Dermatology and Lynderm Research Inc., Markham, ON, Canada; 2 Western University, London, ON, Canada; 3 RBC Consultants, Anneke Andriessen and Co. BV, Malden, Netherlands; 4 University of Toronto, Toronto, ON, Canada; 5 McGill University Health Centre, Montreal, QC, Canada; 6 University of Calgary, Calgary, AB, Canada; 7 Skin Health and Wellness Centre, Dermatology Research Institute, and Probity Medical Research, Calgary, AB, Canada; 8 University of Manitoba, Winnipeg, MB, Canada; 9 SKiNWISE Dermatology, Winnipeg, MB, Canada; 10 Centre de Recherche Dermatologique du Québec, Québec City, QC, Canada

**Keywords:** alopecia areata, Canadian dermatology, consensus, JAK inhibitors, treatment algorithm

## Abstract

**Introduction:**

Alopecia areata (AA) is an autoimmune, inflammatory condition characterized by non-scarring hair loss on the scalp, face, or body. It can significantly affect a person’s quality of life (QoL) and mental health.

**Methods:**

Using a modified Delphi method, a panel of expert dermatologists developed a practical treatment algorithm to help clinicians deliver personalized, safe care to diverse patients with AA.

**Results:**

The approach to the algorithm introduces a broader definition of disease severity, incorporating not only Severity of Alopecia Tool (SALT) scores but also quality-of-life impact and the rate of disease progression.

**Discussion:**

Recent therapeutic advances have expanded access to effective, safe treatments for a broader population of AA patients. Ritlecitinib, a Janus kinase 3/TEC inhibitor, is currently the only oral Janus kinase inhibitor approved for adolescents and adults aged 12 years and older, whereas baricitinib, a Janus kinase1/2 inhibitor, is approved for adults aged 18 years and older. However, questions on the long-term safety of these therapies, access, and personalization still need to be addressed.

**Conclusion:**

Future efforts should validate and refine the algorithm across real-world cohorts and ensure equitable access to personalized treatment for patients with AA.

## 1 Introduction

Alopecia areata (AA) is an inflammatory, relapsing, non-scarring hair loss disorder affecting 2% of the population (1). Manifestations range from localized scalp patches to complete loss of body hair. Hair loss typically presents as bald patches on the scalp, face, or body that may progress to complete loss of scalp hair (Alopecia Totalis (AT) or total loss of all body hair Alopecia Universalis (AU) (2). Nail manifestations, such as nail pitting or trachyonychia, may occur in up to 46% of patients with AA (3, 4). AA pathogenesis results from the collapse of immune privilege and cytotoxic-lymphocyte-mediated damage to hair follicles in susceptible individuals (5). The condition frequently coexists with autoimmune diseases such as thyroid disease, vitiligo, psoriasis, systemic lupus erythematosus (SLE), and rheumatoid arthritis (RA) (1–3). AA is also associated with significant psychosocial impairment and reduced quality of life (QoL) (2). The disease can profoundly affect appearance and self-esteem, leading to emotional distress and high rates of anxiety, depression, and suicidal ideation (5, 6). A summary of key QoL studies in AA is provided in Table 1. Recent advances in Janus kinase (JAK) inhibition have revolutionized AA management. JAK inhibitors (JAKi) are effective in downregulating inflammation at the level of the hair follicle (7–9). However, guidance on incorporating these new therapies and navigating escalation based on QoL and disease dynamics remains limited. This consensus algorithm aims to fill this gap by providing practical, evidence- and experience-based guidance for the management of AA patients aged 12 and older.

**Table 1:** Summary of key quality of life studies and psychological impact of alopecia areata on patients.

Category	Author	Key findings
Psychological	Okhovat et al. (45)	Positive association with anxiety and depression in 6010 patients compared to healthy controls
	Macbeth et al. (46)	Individuals with AA were more likely to develop new-onset depression and anxiety More likely to have time off work certificates, be unemployed and have higher rates of antidepressants
	Bitan et al. (47)	Anxiety is associated with AA across all age groups and genders above 30 years Depression associated with AA, particularly in the 30–49 age group, with a higher association among females A negative association was found between AA and schizophrenia. No association was found between AA and bipolar disease.
Quality of life	Putterman et al. (48)	Significant negative correlations were found between SALT scores and DLQI score
	Jankovic et al. (49)	Impaired QoL in AA patients; however, increased QoL when compared to psoriasis, atopic dermatitis, and onychomycosis
	Velez-Muniz et al. (50)	QoL disturbance was detected in 77.6% of adults with AA, 65.9% had signs of depression or anxiety, and 12.8% were at risk of committing suicide

Alopecia areata (AA), Severity of Alopecia Tool (SALT), quality of life (QoL), Dermatology Life Quality Index (DLQI).

## 2 Materials and methods

This clinical treatment algorithm was developed using a structured, transparent methodology aligned with the AGREE (Appraisal of Guidelines for Research and Evaluation) II framework (10). The development process included the following components:

### 2.1 Study design and setting

This was a consensus-based expert panel initiative conducted in Canada. A multidisciplinary panel of eight Canadian dermatologists convened on November 16, 2024, during the Dermatology Update Conference in Montreal, Quebec.

### 2.2 Panel composition and expertise

Panelists were selected for their clinical expertise in AA, therapeutic innovation, and academic contributions. All had significant experience treating AA in adolescents and adults.

### 2.3 Literature review and evidence synthesis

A structured literature review was conducted by two independent reviewers (AnA, SG) using PubMed as the primary database. This review aimed to address two key clinical questions regarding AA treatment (Table 2). Searches covered English-language publications from January 2015 to July 2024. A secondary search was performed using Google Scholar to capture grey literature and supplementary materials. Keywords included combinations of “alopecia areata” with “minoxidil,” “JAKi,” “corticosteroids,” “immunotherapy,” and other relevant therapies. Of the 72 identified articles, 40 met predefined inclusion criteria that emphasized studies of therapeutic interventions in patients aged ≥12 years. Eligible studies included randomized controlled trials (RCT), systematic reviews, cohort studies, and clinical guidelines. Each study was appraised using a modified GRADE framework, which considered study design, sample size, outcome consistency, and relevance to the target population (11). Graded evidence was used to inform treatment tiering within the algorithm (Table 3).

**Table 2:** Systematic literature review questions.

Systematic literature review questions
Question 1: What is known from published systematic literature searches, meta-analyses, guidelines, consensus papers, and algorithms on the topical and systemic treatment of alopecia areata for patients aged ≥12 years?
Question 2: Which information from published systematic literature searches, meta-analyses, guidelines, and consensus papers on the topical and systemic treatment of alopecia areata for patients aged ≥12 years is relevant for the development of the algorithm?

**Table 3:** Graded evidence for various alopecia areata treatments from systematic literature search with legend.

Recommendation/conditional recommendation	Level of evidence	Type of studies
Topical minoxidil	B3	Comparative studies variable outcomes
Systemic CS for adults with severe AA	C4	Individual cohorts
Cyclosporine monotherapy or combined with systemic CS for adults with severe AA	B3	Systematic review, case–control studies
Azathioprine monotherapy or combined with CS for adults with severe AA	C4	Individual cohorts
Methotrexate monotherapy or combined with CS for adults with severe AA	C4	Case series
Simvastatin/ezetimibe for adults with severe refractory AA	C4	Case series
Inosiplex for adults with severe refractory AA	C4	Individual cohorts
Antihistamine for AA	C3	Case–control studies
Oral JAKi 1/2 for adults with severe AA	A2	individual RCTs
Dupilumab subcutaneously	B3	case–control studies

Grade	Details
A	Clinical double-blind RCT of high quality (e.g., sample-size calculation, flow chart of patient inclusion, intention-to-treat analysis, sufficient sample size)
B	RCT of lesser quality (e.g., only single-blind; limited sample size, but with at least 15 patients per study arm)
C	Comparative trial with severe methodologic limitations (e.g., not blinded, very smal sample size, no randomization)

Grade	Details
1	Further research is unlikely to change confidence in the estimate of effect (i.e., at least two grade A trials are available, and their results are largely consistent with results of additional grade B or grade C studies)
2	Further research is likely to have an important effect on confidence in the estimate of effect and may change the estimate (i.e., at least three grade B trials are available, and their results are largely consistent with any additional grade C trials)
3	Further research is very likely to have an important effect on confidence in the estimate of effect and is likely to change the estimate (i.e., conflicting evidence or limited number of trials, mostly grade B or grade C)
4	Any estimate of effect is very uncertain (i.e., little or no systematic experimental evidence; trials extremely limited in number and/or quality)

Alopecia areata (AA), Corticosteroids (CS), Randomized Controlled trials (RCTs), Janus Kinase Inhibitors (JAKi).

### 2.4 Consensus process

**Figure 1:**
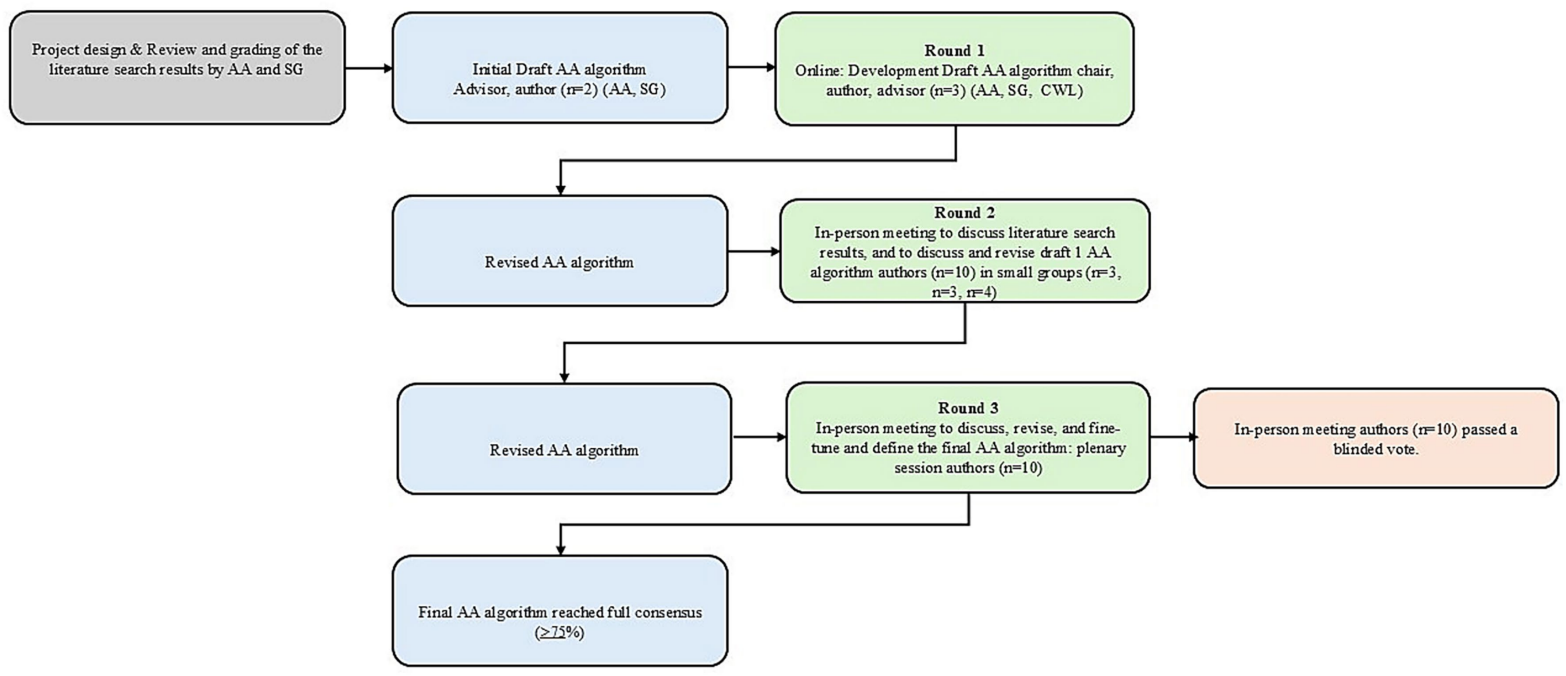
Delphi process.

A modified Delphi technique was employed to develop the treatment algorithm. Panelists participated in multiple structured discussions, beginning with small-group sessions and progressing to a plenary session. Consensus was defined as ≥75% agreement in blinded voting rounds. Items lacking consensus were revised through facilitated discussion and re-evaluated in subsequent rounds (Figure 1).

### 2.5 Ethical considerations and conflict of interest management

All panelists disclosed potential conflicts of interest before participation. The algorithm development was conducted independently of pharmaceutical industry sponsorship. No patient-level data were used, and ethics board review was not required.

### 2.6 Outcome

The final algorithm was refined through iterative online collaboration and includes stratified treatment recommendations for mild, moderate, and severe AA, along with tailored considerations for pediatric, elderly, and comorbid patient populations.

### 2.7 Structured review findings

The literature review yielded 72 eligible publications, of which 40 were selected for full analysis based on predefined inclusion criteria. The included studies comprised 12 RCTs, 5 systematic reviews, 4 meta-analyses, 8 cohort studies, and 11 case series or expert guidelines. The highest-quality evidence (Level A, 1–2) supported the use of oral JAKi —particularly baricitinib, ritlecitinib, and deuruxolitinib—in patients with moderate to severe AA (Table 3). Topical and intralesional corticosteroids were widely represented in lower-level evidence (Level B or C, Grades 3–4), with limited RCT data but extensive real-world use. Contact immunotherapy (e.g., DPCP) and systemic immunosuppressants (methotrexate, cyclosporine) were supported by case series, cohort data, and systematic reviews with variable outcomes. Oral minoxidil and topical JAKi were less frequently studied but showed emerging promise, particularly in combination regimens. Studies addressing psychosocial outcomes and QoL were limited but highlighted the burden of disease and informed the decision to upgrade severity for high-impact presentations. The algorithm was thus anchored in high-level data where available and supplemented by clinical consensus in domains where evidence was heterogeneous or evolving.

## 3 Results

### 3.1 The algorithm

**Figure 2:**
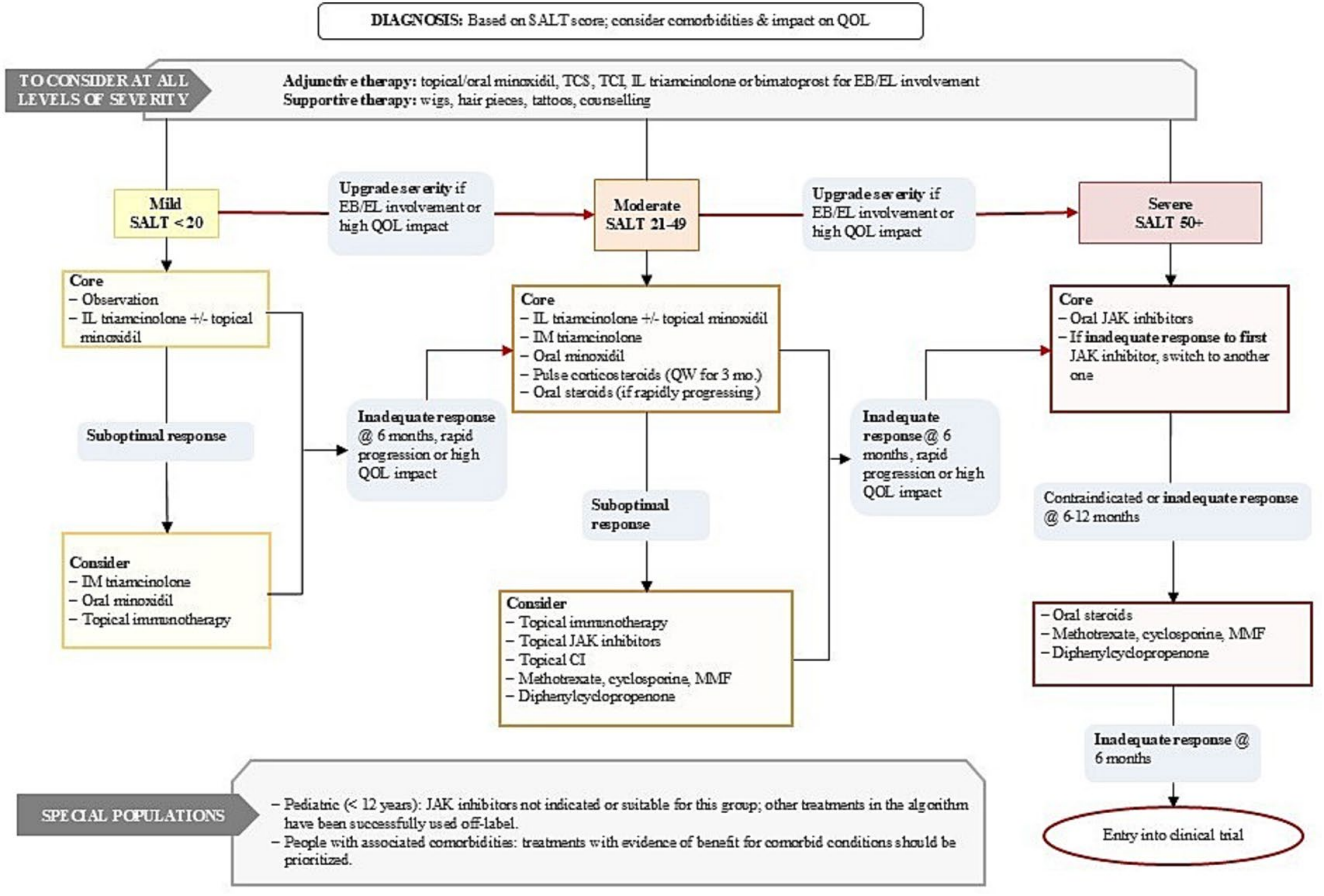
A practical algorithm for the treatment of alopecia areata for ages 12 + years. EB, Eyebrows; EL, Eyelashes; IL, Intralesional; IM, Intramuscular; MMF, Mycophenolate mofetil; QOL, Quality of life; SALT, Severity of Alopecia Tool; TCS, Topical corticosteroids; TCI, Topical calcineurin inhibitors.

Considering SALT scores, comorbidities, disease progression rate, and impact on QoL measures, the algorithm is designed to help clinicians identify the most appropriate treatment strategy for each patient with AA (Figure 2).

*Severity of Alopecia Tool Score:* The SALT score quantifies the percentage of scalp hair loss across four quadrants to assess disease severity (Table 4) (12, 13). A modified version of the SALT score, the AA scale, increases severity by one level in the presence of eyebrow or eyelash involvement, significant psychosocial impact, rapidly progressing AA, or insufficient treatment response after at least 6 months of therapy (13). Major limitations of the SALT scoring system include high interrater variability and inclusion of other types of alopecia, such as androgenic alopecia, which challenge the consistency and standardization of its use (12).

**Table 4:** Severity of alopecia tool (SALT) score (12).

Alopecia areata scale	
Scalp hair loss	
Severity	Extent of scalp hair loss
Mild AA	20% or less scalp hair loss
Moderate AA	21–49% scalp hair loss
Severe AA	50–100% scalp hair loss
If mild to moderate, increase AA severity rating by 1 level or more of the following is present: • Negative impact on psychosocial functioning resulting from AA • Noticeable involvement of eyebrows or eyelashes • Inadequate response after at least 6 months of treatment • Diffuse (multifocal) positive hair pull test consistent with rapidly progressive AA	

Alopecia areata (AA), Severity of Alopecia Tool (SALT). Adapted from King et al. (12).

*Best-Practice Treatment Recommendations: For each AA severity category (mild, moderate, severe), the algorithm outlines best-practice “core” treatment options, along with “consider” treatment options when the core therapy yields a suboptimal response*. Adjunctive and supportive therapies are recommended across all stages of AA. Treatment response is primarily assessed by improvement in SALT score at 6-month intervals. In the event of an inadequate response, the algorithm recommends escalation to the next severity tier (Figure 2). Experts agreed that an inadequate response would be a decision made by the patient and clinician, as response to therapy can be variable. Supportive therapies such as wigs/hairpieces, tattoos, and psychological counseling are essential to help patients manage the psychosocial burden of the disease.

### 3.2 Mild AA

Mild AA (SALT <20%) core treatment options include topical and intralesional corticosteroids (ILC); triamcinolone acetonide with or without topical minoxidil. In cases of insufficient response, intramuscular corticosteroids (triamcinolone acetonide), oral minoxidil, or topical calcineurin inhibitors (e.g., tacrolimus) may be considered. Intralesional corticosteroids (ILCs) are the most frequently used treatments in clinical practice; however, no randomized controlled trials (RCTs) have evaluated their efficacy in AA (14). Intramuscular corticosteroids (triamcinolone acetonide) has demonstrated efficacy in refractory AA, with a 63% response rate in a retrospective study of 27 AA patients (age range 12–60 years) (15). Systemic corticosteroids are supported mainly by individual cohort studies. Similarly, minoxidil has limited evidence. A meta-analysis of six RCTs showed that topical minoxidil (1, 3, and 5%) was more effective than placebo during short-term follow-up of under 6 months, although several studies reported limited efficacy (16–18). Oral minoxidil (2.5 mg daily) has shown promise, especially when combined with oral JAKi such as tofacitinib (19–21).

### 3.3 Moderate AA

Moderate AA (SALT 21–49%) core treatment options include topical corticosteroids and ILC, topical and oral minoxidil, intramuscular corticosteroids (triamcinolone acetonide), and systemic corticosteroids (oral prednisone or pulse IV methylprednisolone). Pulse corticosteroids (methylprednisolone 500 mg/day IV) have shown favorable efficacy, particularly in recent-onset AA, but are less effective in patients with disease duration beyond 6 months (22). For non-responders, additional treatments include topical contact sensitizers (e.g., diphenylcyclopropenone [DPCP]; squaric acid dibutyl ester [SADBE]), topical JAKi (e.g., 1% ruxolitinib or 2% tofacitinib), topical calcineurin inhibitors, and oral immunosuppressants (methotrexate, cyclosporine, azathioprine, or mycophenolate mofetil) (Figure 2). In mild-to-moderate AA, topical therapies like contact sensitizers and calcineurin inhibitors offer moderate efficacy, while topical JAKi serve as alternatives to topical, intralesional, and systemic corticosteroids (23). Oral immunosuppressants are supported primarily by case series or case–control studies (Table 3). A systematic review of 14 case studies found IV methylprednisolone combined with oral cyclosporine to be more effective than oral cyclosporine alone (24). Phan et al. reported over 50% response rates with oral methotrexate in a meta-analysis (25). However, immunosuppressants carry significant risks, including renal/hepatic toxicity and malignancy, limiting their widespread use (26).

### 3.4 Severe AA

The core treatment option for severe AA (SALT >50%) are the oral JAKi such as baricitinib (JAKi1/2), and ritlecitinib (JAKi3/TEC) (Table 5) (26). Off-label use of oral tofacitinib (JAKi1/2/3) and oral upadacitinib (JAKi1) has also been documented in the literature. Deuruxolitinib (oral JAKi1/2) has also shown efficacy in the THRIVE-AA1/AA2 double-blinded, placebo-controlled phase 3 clinical trials; however, it is not yet commercially available (27, 28). Lastly, IV pulse corticosteroids, methotrexate and cyclosporine may also be used in severe AA (26). Joly et al. demonstrate that methotrexate in combination with low-dose prednisone leads to complete hair recovery in 31% of patients with AT or AU by month 12 (29).

**Table 5:** JAK inhibitors for alopecia areata.

JAK inhibitor	Selectivity	Key pivotal trial data	% achieving SALT ≤20	Key features
Approved				
Ritlecitinib	JAK3/TEC	ALLEGRO Phase 2b/3	23% at week 24 (50 mg daily)	Only JAK inhibitor approved for ages 12 years and older
Baricitinib	JAK1/2	BRAVE-AA1	38.8% (4 mg at week 36) BRAVE-AA1	First JAK inhibitor approved for AA Two available dosages: 2 mg and 4 mg
			35.9% (4 mg at week 36) BRAVE-AA2	
		BRAVE-AA2		
Off-label				
Tofacitinib	JAK1/2/3	Phase 2, open-label	32% achieved ≥50% in SALT score by week 24 (tofacitinib 5 mg twice daily)	Pan-JAK inhibitor, also approved for rheumatoid arthritis, psoriatic arthritis, ulcerative colitis, juvenile idiopathic arthritis, and ankylosing spondylitis
Ruxolitinib	JAK 1/2	Small trials, case reports	----	Used in topical form
In the pipeline				
Brepocitinib	Dual TYK2/JAK1 inhibitor	Phase 2a (NCT 04797650) currently in trial	64% at week 24 ** achieved 30% improvement in SALT score	First TYK2/JAK1 inhibition combination

**All JAK inhibitors carry a black box warning for serious infections, malignancies, and thrombosis. Alopecia areata (AA), Severity of Alopecia Tool (SALT), Janus Kinase Inhibitors (JAKi), Baricitinib for Alopecia Areata study (BRAVE-AA), Pseudokinase2 (TYK2) (protects against multiple autoimmune diseases).

The pivotal baricitinib AA clinical trials, BRAVE-AA1 and BRAVE-AA2, demonstrated that approximately >35% of patients being treated with 4 mg daily baricitinib achieved a SALT score ≤20 by week 36 of treatment (30). Ritlecitinib is the only oral JAKi approved for use in both adults and adolescent patients with severe AA (31). It irreversibly inhibits JAK3 and TEC kinase activity which inhibits the downstream release of pro-inflammatory AA-driving cytokines and cytolytic activity of lymphocytes (31, 32). ALLEGRO-2b/3, the phase 2b/3 pivotal ritlecitinib study demonstrated that 23% of severe AA patients (SALT>50%) achieved a SALT ≤20 at week 24 on 50 mg daily of ritlecitinib treatment compared to 1.6% in the placebo group (31). 31% of severe AA patients on 200 mg ritlecitinib loading dose followed by 50 mg daily achieving a SALT score of 20 or less at week 24 (31). At week 48, the patients continued to improve with 43% of patients achieving SALT score ≤20 (31). The safety of ritlecitinib was evaluated in 3 randomized, placebo-controlled clinical studies and one long-term study in patients with AA (31, 33). The most common adverse reaction reported in ≥1% of patients in the ritlecitinib-treated group was headache, diarrhea, and acne, which had a similar incidence in the placebo groups (33). Across all clinical trials, a total of 1628 subjects were treated with ritlecitinib; there was no signal (reported in >1% of subjects and at a higher rate than placebo) for malignancy, thrombosis, or MACE (33). There was also 1 case of decreased lymphocyte counts (absolute lymphocyte count <500/mm^3^) and 1 case of decreased platelet counts (<100,000/mm^3^) (31). While patients should be monitored on ritlecitinib, the long-term safety studies demonstrated a favorable safety profile. Pooled safety data from BRAVE-AA1 and BRAVE-AA2 trials also did not reveal significant safety concerns for baricitinib (34). The most frequent adverse events related to baricitinib were acne and elevated creatine kinase (CK) levels (34). There was a low rate of serious infection and only one MACE, a pulmonary embolism event, and 3 malignancies across 1303 adults and 4 years of treatment. Longer observation periods and longer-term patient exposures will also be essential to ascertain risk. If there is an inadequate response to one oral JAKi, the algorithm suggests switching to another oral JAKi or reverting to oral corticosteroids/immunosuppressants. In addition, oral minoxidil can be considered as adjuvant therapy. The “consider” treatment options for severe AA should also be used for patients for whom oral JAKi is contraindicated. If patients remain refractory to all treatments proposed in the algorithm, experts recommend entry into a clinical trial (see *Emerging Treatments* section).

## 4 Treatment considerations for special Sites & Populations

Each AA treatment must be tailored to the specific patient, taking into account disease severity, comorbidities, and therapy goals. Oral JAKi such as ritlecitinib and baricitinib have shown the greatest efficacy and practicality in regrowth of eyelashes, eyebrows, and nose hairs, as well as normalization of nails. The eyebrow and eyelash response in ALLEGRO-2b/3 was significant, with 29 and 28.9% of patients receiving 50 mg daily of ritlecitinib showing improvement in eyebrow and eyelash regrowth, respectively, by week 24, compared to 4.7 and 5.2% of patients receiving placebo (31). In the BRAVE-AA1 and BRAVE-AA2 trials, baricitinib 4 mg demonstrated full eyebrow regrowth or minimal gaps in 35.2% (AA1) and 38.9% (AA2) of patients and full eyelash regrowth or minimal gaps in 36.2% (AA1) and 36.8% (AA2) of patients (30). Topical JAKi preparations have also demonstrated efficacy in eyelash regrowth with twice daily application (35). This can be a good alternative for pediatric/adult patients or patients who cannot take oral JAKi.

In general, topical therapies (such as corticosteroids and contact sensitizers) as well as ILCs have the highest level of evidence for use in pediatric AA. The safety profile of long-term oral JAKi use has been the main concern of their use in pediatric and geriatric populations. JAKi are labeled with a black box warning for serious infections, mortality, malignancy, thrombosis, and MACE (33, 36). The warning is inherited from clinical trials investigating the use of pan-JAKi, tofacitinib, in a population of RA patients aged>50 years with existing cardiovascular risks, which demonstrated an increased risk of MACE, malignancies (lung cancer and lymphoma), and venous thromboembolism (particularly with the 10 mg dose) (36). The risks were shown to be dose-dependent and more significant than the risk of adverse events in patients on TNF-inhibitors (36). However, large clinical trial data of more recent JAKi have not reflected a significant increase in these events. JAKi should not be used in pregnant women (32). Experts agree that JAKi should also be used with caution in patients with a history of malignancy, thromboembolic events, or over the age of 65 until more safety data becomes available.

Pediatric AA is often more severe with a worse prognosis than adult-onset AA; thereby, warranting a rapid therapeutic option to prevent disease progression and the detrimental impact on a child’s development/QoL (37). While a handful of case reports exist in the literature for off-label use of oral tofacitinib in pediatric AA, ritlecitinib is the only JAKi approved for adolescents aged ≥12 years, thereby making it a good therapeutic option for treatment in the younger patients (37). Comorbidities must also be considered prior to starting an oral JAKi. For example, JAKi such as oral tofacitinib have been associated with a higher rate of all-cause mortality in patients with RA, making healthcare providers often cautious in using JAKi in patients with RA. However, ritlecitinib has been studied in a small subset of RA patients in a phase 2 clinical trial, whereby it did not demonstrate any serious treatment-related adverse events over an 8-week treatment period (38). Ritlecitinib is not approved for RA to date (33).

In addition, patients with comorbid nonsegmental vitiligo may benefit from ritlecitinib treatment, as phase 2b clinical trials demonstrated that ritlecitinib was also effective and well-tolerated in patients with vitiligo (26). Ritlecitinib should not be used in female patients who are pregnant or breastfeeding (33). Use of ritlecitinib and other oral JAKi in the geriatric population may also be considered; however, the risks and benefits of JAKi must be weighed along with a patient’s comorbidities.

### 4.1 Emerging treatments

Treatments for AA have seen rapid growth and increasing availability in recent years. In addition to the recent approval of ritlecitinib for AA, brepocitinib (a TYK2/JAK1 inhibitor) is currently undergoing clinical trials and has shown promising efficacy in AA and other conditions, including psoriatic arthritis, plaque psoriasis, ulcerative colitis, and hidradenitis suppurativa (9). Other JAKi, such as upadacitinib, have also been evaluated in Phase 3 clinical trials for severe AA. Deuruxolitinib (JAK1/2 inhibitor) has also been shown efficacy in severe AA patients and has been FDA approved since July 2024 (27, 39). THRIVE-AA1/THRIVE-AA2 clinical trials demonstrate that 29.6 and 33% of patients on 8 mg deuruxolitinib twice daily and 41.5 and 38% of patients on 12 mg deuruxolitinib twice daily achieved SALT ≤ 20 at week 24 in THRIVE-AA1 and THRIVE-AA2, respectively (27, 39).

Additionally, dupilumab, an IL-4/IL-13 blocker, has been investigated for use in AA, based on serum biomarker studies revealing systemic Th1/Th2/IL-12/IL-23 dysregulation in AA patients (40, 41). In a Phase 2b clinical trial, dupilumab demonstrated a SALT 50 improvement in 22.5% of patients after 48 weeks of treatment (40, 42). This suggests that dupilumab may be an appropriate treatment for certain AA patients, particularly those with concomitant type 2 comorbidities such as atopic dermatitis, asthma, and chronic rhinosinusitis with nasal polyps, and thus a Th2/IL-12/IL-23 axis skew (40, 42). Additionally, novel biologics such as the anti-OX40 monoclonal antibodies (IMG-007 and amlitelimab) and anti-IL-7R antibody (bempikibart/ADX-914) are under investigation (2, 43, 44). The development of monoclonal antibodies and small molecules for AA continues to expand, offering hope for more personalized and effective treatment options.

## 5 Limitations

This algorithm provides a broad guide for clinicians to refer to in guiding them in selecting initial treatment for patients 12 years and older with alopecia areata. However, the Delphi consensus meeting did not address long-term management, de-escalation of therapy, or relapses. In the future, it will be important to develop an algorithm for the long-term management of patients on JAKi that addresses potential comorbidities, treatment duration, and drug-monitoring strategies. Future discussions will also include recommendations about beard alopecia, as this was not discussed in this expert panel. The scope of this algorithm was limited to initial treatment selection.

## 6 Conclusion

AA is a prevalent, heterogeneous disease that can be profoundly debilitating. It affects diverse patient populations across ages and ethnicities. Novel targeted agents such as JAKi have revolutionized the treatment paradigms. Despite this, questions remain around long-term safety, access, and personalization. This consensus-based algorithm integrates SALT scoring, disease progression and psychosocial burden to guide clinicians in making individualized treatment decisions. Future efforts should aim to validate and refine the algorithm across real-world cohorts and ensure equitable access to care.
